# Conservative Treatment of Sigmoid Diverticulum Perforation Secondary to Migrated Biliary Plastic Prostheses Inserted by Endoscopic Retrograde Cholangiopancreatography: A Case Report of an Unusual Adverse Event and Literature Review

**DOI:** 10.7759/cureus.79042

**Published:** 2025-02-15

**Authors:** André Orsini-Ardengh, Anna Carolina Orsini-Arman, Bruna Haueisen Figueiredo Zwetkoff, Otavio Micelli-Neto, Rodrigo Cañada T Surjan, Jose C Ardengh

**Affiliations:** 1 Gastrointestinal Endoscopy, Hospital Das Clínicas da Faculdade De Medicina da Universidade De São Paulo, São Paulo, BRA; 2 Gastroenterology, Pontifical Catholic University of Campinas, Campinas, BRA; 3 Digestive Endoscopy, Hospital Moriah, São Paulo, BRA; 4 Surgery, Faculdade De Medicina da Universidade De São Paulo, São Paulo, BRA; 5 Surgery, Hospital Nove De Julho, Diagnósticos da América S.A., São Paulo, BRA; 6 Gastrointestinal Endoscopy, Hospital Das Clínicas De Ribeirão Preto, Ribeirão Preto, BRA; 7 Image Diagnosis, Universidade Federal De São Paulo, São Paulo, BRA

**Keywords:** adverse events, biliary plastic prostheses, biliary stent, complication, intestinal, migrated stent, migration, perforation, stent, stents

## Abstract

Distal migration of biliary plastic stents is rare. Although these stents are primarily used in the treatment of benign diseases of the biliopancreatic tract, their distal migration can lead to severe complications, such as perforation of any part of the digestive system. The authors report a case of sigmoid diverticulum perforation caused by the migration of a biliary plastic stent, which had been initially placed due to a failure to extract a common bile duct (CBD) stone. A review of similar cases in the literature was conducted, and the findings were analyzed in relation to the reported case. The search was performed in MEDLINE and the Cochrane Library, covering studies published between 1975 and 2025. Only studies describing the placement of biliary plastic stents during endoscopic retrograde cholangiopancreatography (ERCP) were included, while studies with incomplete data were excluded. This study highlights this rare and serious complication, which carries a high morbidity rate. Despite careful stent positioning during ERCP and periodic follow-up, this adverse event (AE) cannot always be prevented. Although distal stent migration with perforation can often be treated endoscopically, preoperative evaluation of the patient's clinical condition and precise localization of the perforation is crucial for successful endoscopic stent removal, thus avoiding the need for surgery.

## Introduction

The biliary plastic prostheses (PP), inserted during endoscopic retrograde cholangiopancreatography (ERCP), are used to treat benign and/or malignant biliary obstructions [1]. Common indications include bile leakage after cholecystectomy, failure to extract common bile duct (CBD) stones, post-cholecystectomy inflammatory biliary stenosis, post-liver transplant anastomosis biliary duct stricture (BDS), and pseudotumor chronic pancreatitis (PCP). On the malignant spectrum, pancreatic cancer (PC), cholangiocarcinoma, liver cancer, and hepatic metastases are among the conditions treated with PP [1,2].

Adverse events (AEs) occur in 5% of cases after PP insertion. These events are usually immediate and unrelated to the type of prostheses, which may include infections, hemorrhage, and pancreatitis. Late AEs include PP dysfunction, acute cholecystitis (AC), duodenal perforation, ulceration, and bleeding. Migration occurs in approximately 6% of PP cases, 1% of partially covered self-expandable metallic stents (SEMS), and 20% of fully covered SEMS. PP migration is more frequent in benign obstructive conditions, with endoscopic treatment being feasible in over 90% of cases with low morbidity [3,4].

Secondary perforation due to PP migration primarily affects the duodenum during insertion or late migration [5]. Other reported sites of perforation include the colon, distal ileum, liver, and pancreas. Diverticular disease of the colon increases the risk of secondary perforation from migrated PP [6,7]. Most cases require surgical procedures for PP removal, repair and suturing of the perforated organ(s), or resection of the affected colonic segments. However, the feasibility of endoscopic removal following distal migration of a PP with colon perforation depends on factors such as clinical stability, absence of peritoneal irritation, and availability of a surgical team for eligible patients [7,8]. A recent systematic review on this topic revealed that the global mortality rate was 17.4%, making this AE a concern regarding the treatment approach [9].

We report a case of sigmoid diverticular perforation caused by PP migration after the failed extraction of a giant choledocholithiasis. The clinical scenario of a perforated acute abdomen, the time between PP insertion and symptom onset, and the successful conservative treatment with PP removal via colonoscopy make this case noteworthy. Furthermore, the authors conducted a systematic review of reported cases of colon perforation caused by PP migration.

## Case presentation

A 77-year-old female patient was admitted to the emergency room with abdominal pain localized in the left flank and iliac fossa, with no signs of peritoneal irritation, afebrile, and hemodynamically stable. Medical history revealed chronic diseases such as diabetes, hypertension, hypothyroidism, and obesity. The patient underwent cholecystectomy in 1990, due to calculous gallbladder disease. In 2009, she presented with mild jaundice with increased direct bilirubin levels (1.9 mg/dL), elevated alkaline phosphatase (777 U/L), gamma-glutamyl transpeptidase (1666.0 U/L), pyruvic transaminase (288 U/L), and oxaloacetic transaminase (264 U/L). At that time, abdominal ultrasonography (US) and magnetic resonance cholangiopancreatography (MR/MRCP) showed gallstones in the proximal third of the CBD with upstream ductal dilatation. On that occasion, an ERCP was performed to remove stones. Two years later she had a new episode of jaundice and underwent another ERCP, which showed saccular dilatation of the common hepatic duct with filling defects inside. Ductal screening with balloon extractor, stone removal, and placement of a polyethylene biliary stent (8.5 Fr x 9 cm) were performed.

**Figure 1:**
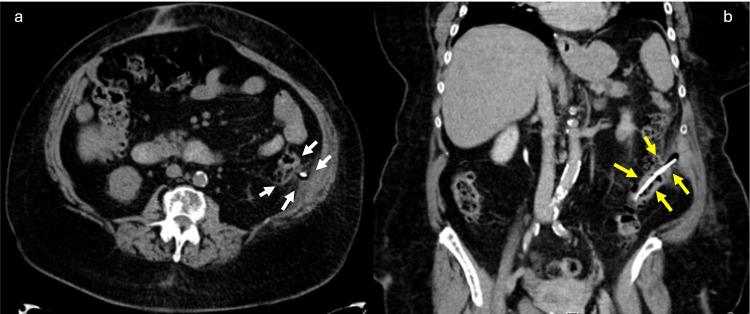
(a) Abdominal axial CT scan showing a tubular structure in the lumen of the sigmoid colon, extending to the peritoneal membrane on the left abdominal wall (white arrows). (b) Coronal section showing a plastic biliary stent, extending to the peritoneal membrane on the left abdominal wall (yellow arrows).

The clinical history and physical examination supported the presumptive diagnosis of acute pancreatitis (AP). Computed tomography for emergency assessment showed a tubular structure with hyper attenuation, located in the lumen of the sigmoid. The upper end of the stent transfixed the colon and peritoneal membrane, reaching the left abdominal wall with no signs of peritonitis (Figure 1).

**Figure 2:**
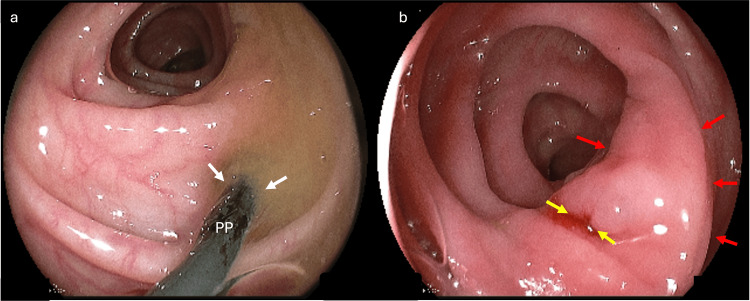
(a) Biliary PP transfixing diverticulum with purulent discharge in the sigmoid colon (white arrow). (b) Diverticular ostium after PP stent removal (yellow arrow), and peripheric edematous region (red arrows). PP, plastic prostheses

We opted for PP stent removal by colonoscopy despite the exuberant clinical setting of abdominal pain (Figure 2). The procedure was uneventful, with satisfactory patient recovery without surgical intervention. Intravenous ceftriaxone and metronidazole have been administered since her admission. She stayed in the hospital for three days after the prosthesis removal, with a good outcome and no pain complaints.

Literature review

Method

Population design: We included all patients who underwent ERCP with PP placement for the treatment of benign and/or malignant biliary and pancreatic diseases. Case reports with complete data regarding the occurrence and outcomes of perforation, as well as the therapeutic strategy employed, were selected. 

Database and search strategy: The search was conducted in MEDLINE and the Cochrane Library for studies published between 1975 and 2025. Medical Subject Headings (MeSH) terminology and user-defined keywords commonly found in relevant articles were applied. The keywords included "(biliary plastic prostheses OR biliary stent OR stent OR stents) AND (migration OR migrated OR complication OR adverse events OR perforation, intestinal) AND (colon perforation OR colonic perforation OR sigmoid perforation OR caecum perforation OR appendiceal perforation OR diverticular impaction OR colovesical fistula)."

Only publications involving human subjects were included, and the bibliographies of relevant articles were reviewed to identify additional studies. We primarily identified case series related to this subject. 

Data analysis and outcomes: Data was collected using a predesigned form (Excel spreadsheet) and considered for extraction when available in the text, tables, and/or figures, including information regarding treatment intent. The extracted data included the following categorical variables: epidemiological characteristics of the population (sex and age), indications and diagnoses (benign and malignant) for ERCP, performance of papillotomy, type of PP implanted (diameter and length), time between prosthesis placement and the onset of symptoms related to perforation, location of the perforation, and the therapeutic strategy adopted. Studies in which we did not find data we considered important, such as indication for ERCP, perforation site, and type of treatment administered, were excluded.

Results

**Figure 3:**
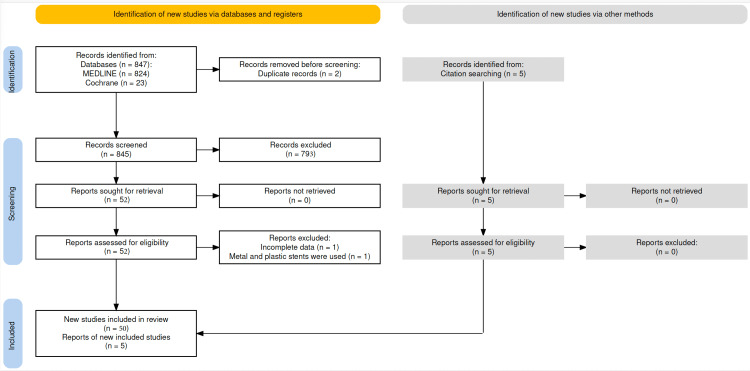
PRISMA flowchart of the search strategy for included and excluded cases.

Study population: This study included all case reports identified through the search strategy described in the previous subsection, in addition to the description of the current case. The results of the search strategy can be seen in Figure 3. 

The search found 52 studies, of which two were excluded. One for describing the migration of a biliary pigtail PP associated with a biliary SEMS [10] and another for presenting incomplete data [11]. Another five reports were cited by other studies [12-16]. Therefore, 55 studies were identified, reporting a total of 58 patients, with Hogendorf et al. [15], Ruffolo et al. [17], and Virgilio et al. [18] reporting two cases each. Including the present case, 59 cases were analyzed [19]. The average age was 69.7 years (ranging from 26 to 94), with a higher prevalence in women: 40 out of 59 (68%) presented with colonic perforation due to the distal migration of the plastic biliary prosthesis inserted during ERCP. The clinical characteristics of the population are presented in Table 1.

**Table 1:** Clinical features of indications for ERCP and the type, diameter, and length of the biliary PP. ABS, anastomotic bile stricture; AC, acute cholangitis; B, benign; BDS, bile duct strictures; BL, bile leakage; CBD, common bile duct; F, female; JDD, juxtapapillary duodenal diverticulum; LC, laparoscopic cholecystectomy; LT, liver transplantation; M, male; M, malign; N/A, not available; PC, pancreatic carcinoma; PCP, pseudotumoral chronic pancreatitis; PL, primary pancreatic lymphoma; SF, splenic flexure; PP, plastic prostheses

N	Author, publication year	Gender	Age	Benign disease?	Type of lesion	Papilotomy (yes/no)	PP (diameter (F))	PP (length (cm))
1	Ruffolo TA et al., 1992 [17]	F	72	Yes	CBD stone	Yes	10	12
2	Ruffolo TA et al., 1992 [17]	F	70	Yes	CBD stones + AC	Yes	7	5
3	D’Costa H et al., 1994 [20]	M	73	No	PC	Yes	10	12.5
4	Schaafsma RJ et al., 1996 [21]	F	77	Yes	CBD stone	Yes	7	10
5	Baty V et al., 1996 [22]	F	86	No	PC	Yes	10	10
6	Lenzo NP and Garas G 1998 [23]	F	85	Yes	CBD stone	Yes	10	7.5
7	Ang BK et al., 1998 [24]	M	71	Yes	CBD stones + AC	Yes	10	12
8	Storkson RH et al., 2000 [25]	M	86	Yes	JDD + CBD dilatation	Yes	7	5
9	Figueras RG et al., 2001 [26]	M	47	Yes	PCP + BDS	Yes	10	10
10	Klein U et al., 2001 [27]	M	70	Yes	BDS after LC	Yes	10	10
11	Wilhelm A et al., 2003 [28]	F	85	Yes	CBD stone	Yes	7	10
12	Elliott M et al., 2003 [29]	F	80	Yes	CBD stone	Yes	10	10
13	Diller R et al., 2003 [30]	F	58	Yes	LT + ABS	Yes	7	10
14	Blake AM et al., 2004 [31]	F	65	Yes	CBD stone	Yes	N/A	N/A
15	Soto-Avila JJ et al., 2006 [32]	F	47	Yes	CBD stones + BDS	Yes	7	10
16	Anderson EM et al., 2007 [33]	F	80	Yes	CBD stone	Yes	10	7
17	Namdar T et al., 2007 [34]	F	65	Yes	BL after LC	No	12	10
18	Belyaev O et al., 2008 [35]	F	N/A	Yes	CBD stone	N/A	N/A	N/A
19	Aryal KR et al., 2008 [36]	F	57	Yes	BL after LC	Yes	10	7
20	Hunter K et al., 2010 [12]	F	72	Yes	BDS after LC	Yes	10	10
21	Bagul A et al., 2010 [37]	F	79	Yes	BDS after LC	Yes	10	9
22	Wagemakers S et al., 2011 [38]	F	76	Yes	CBD stone	Yes	N/A	N/A
23	Peter A et al., 2011 [39]	F	69	Yes	BDS after LC	Yes	10	7
24	Jafferbhoy SF et al., 2011 [40]	F	82	Yes	BL after LC	Yes	7	7
25	Lankisch TO et al., 2011 [41]	F	65	No	PC	Yes	10	10
26	Malgras B et al., 2011 [42]	M	73	No	PC	Yes	10	5
27	Alcaide N et al., 2012 [43]	M	73	Yes	CBD stones + BDS	Yes	10	12
28	Kittappa K et al., 2013 [44]	F	58	Yes	CBD stones + BDS	Yes	10	12
29	Jones M, et al., 2013 [45]	M	66	Yes	CBD stones + BDS	Yes	10	8
30	de Andres AB et al., 2013 [13]	M	70	Yes	CBD stone	Yes	10	9
31	Warnock JM et al., 2013 [14]	F	77	No	PL	Yes	10	8
32	Barut I and Tarhan OR, 2014 [46]	F	26	Yes	BDS after LC	Yes	10	10
33	Konstantinidis C et al., 2014 [47]	F	69	Yes	CBD stone	Yes	N/A	N/A
34	Virgilio E et al., 2015 [18]	F	N/A	Yes	CBD stone	N/A	N/A	N/A
35	Virgilio E et al., 2015 [18]	F	N/A	Yes	CBD stone	Yes	10	12
36	Mady RF et al., 2015 [48]	M	N/A	No	PC	N/A	N/A	N/A
37	Chittleborough TJ et al., 2016 [49]	M	73	Yes	CBD stones + AC	Yes	10	5
38	Chou, ND et al., 2017 [50]	F	85	Yes	CBD stone	N/A	N/A	N/A
39	Siaperas P et al., 2017 [51]	F	75	Yes	CBD stone	Yes	10	7
40	Hogendorf P et al., 2018 [15]	F	76	Yes	CBD stone	N/A	N/A	N/A
41	Hogendorf et al., 2018 [15]	M	68	No	PC	Yes	7	10
42	Riccardi, M et al., 2019 [52]	F	79	Yes	CBD stone	Yes	10	10
43	Hnaris K and Bechara R, 2019 [53]	F	90	Yes	Mirizzi's syndrome	Yes	10	10
44	Marcos P et al., 2020 [54]	F	65	Yes	CBD stone	Yes	10	5
45	Tao Y and Long J, 2021 [55]	M	54	Yes	CBD stones + AC + AP	N/A	N/A	N/A
46	Pengemä P et al, 2021 [56]	F	66	Yes	PCP + BDS	Yes	10	5
47	Park TY et al., 2021 [57]	M	74	Yes	CBD stones + AC	Yes	10	7
48	Ong XZ and Leow Y, 2021 [16]	M	57	Yes	CBD stone	N/A	N/A	N/A
49	Yamaguchi D et al., 2022 [58]	F	86	Yes	CBD stones + AC	Yes	7	7
50	Kodia K et al., 2022 [59]	F	60	No	GC + CBD invasion	N/A	N/A	N/A
51	Kwong E et al., 2023 [60]	F	94	Yes	CBD stone	Yes	10	7
52	Rybinski F et al., 2023 [61]	M	78	Yes	CBD stones + AC + AP	Yes	10	10
53	Mohammadi Tofigh A et al., 2023 [62]	F	65	Yes	CBD stone	Yes	10	10
54	Berdugo Hurtado F et al., 2023 [63]	M	50	Yes	BDS after LC	Yes	10	7
55	He Y et al., 2024 [64]	M	35	Yes	CBD stone	Yes	8.5	10
56	Vergara-Fernándes O et al., 2024 [65]	M	67	No	PC	Yes	10	8
57	Swied MY et al., 2024 [66]	F	54	YES	BDS after LC	Yes	7	10
58	Beloy JB et al., 2024 [67]	F	79	Yes	CBD stones + AC	Yes	8.5	7
59	Ardengh AO et al., 2025	F	77	Yes	CBD stone	Yes	8.5	9

Indication for ERCP and sphincterotomy: Fifty out of 59 (85%) patients had benign findings involving the biliary and/or pancreatic ducts, while nine out of 59 (15%) had malignant diseases. All were treated with PP insertion during ERCP. Among the benign findings, the diagnoses included BDS (14), biliary fistula after laparoscopic cholecystectomy (3), choledocholithiasis (36), and a large juxtapapillary diverticulum with CBD dilation (1). Among the identified malignant diseases, 7/59 (12%) were cases of PC, 1/59 (2%) was a primary pancreatic lymphoma, and 1/59 (2%) was a gallbladder carcinoma with CBD invasion. Eleven out of 59 (22%) patients had CBD stricture after cholecystectomy (4/59 (7%) of whom had associated stones), 2/59 (3%) had distal bile duct stricture due to pseudotumoral chronic pancreatitis (PCP), and 1/59 (2%) had a bile duct-to-bile duct anastomosis stricture after liver transplantation. In 36/59 (61%) cases, the authors reported difficulty clearing the bile duct due to the presence of stones, leading to the implantation of PP in 36/59 (61%). Of these, 7/59 (12%) had traumatic bile duct stricture, and 8/59 (13.5%) had acute cholangitis (AC), in which 1/59 (2%) also presented with pancreatitis. Three out of 59 (5%) underwent ERCP with PP insertion due to bile leakage through the cystic duct after cholecystectomy. Sphincterotomy before PP insertion was described in 51/59 (86%) and was performed in 50/51 (98%) patients.

Diameter and length of PPs and migration time: Details regarding the characteristics of the PPs were described in 48/59 (81%) cases. A 10F PP was inserted in 34 out of 48 (71%) cases, a 7F in 10 out of 48 (21%), an 8.5F in three out of 48 (6%), and a 12F in one out of 48 (2%). The length of the PPs was reported as 5 cm, 7 cm, 7.5 cm, 8 cm, 9 cm, 10 cm, 12 cm, and 12.5 cm in 6/48 (13%), 10/48 (21%), 1/48 (2%), 3/48 (6%), 3/48 (6%), 19/48 (40%), 5/48 (10%), and 1/48 (2%) case, respectively. A 10F and 10 cm BPP was implanted in 11/48 (23%) of the cases.

The migration time of the PP was expressed in days and referred to the period between PP implantation during ERCP and the onset of symptoms due to perforation. This analysis was possible in 53/59 (90%) cases. The average time reported was 274.4 days (min. 4 days, max. 2920 days).

Perforation site: The perforation site was described in all reports. The appendix, caecum, ascending colon, sigmoid, and rectum were the sites where PPs were found via endoscopy and/or surgery in 2/59 (3.4%), 4/59 (6.8%), 10/59 (17%), 41/59 (69%), and 2/59 (3.4%) cases, respectively. The sigmoid was the most affected segment, and in 23/59 (39%) patients, the perforation occurred at a diverticulum where the PP had impacted. Overall, 24/59 (40.6%) patients had perforation and PP impaction at a diverticulum, of which 23/59 (39%) in the sigmoid colon and 1/59 (1.7%) in the ascending colon.

Major complications during and after perforation: Major complications during perforation included 3/59 (5.1%) pelvic abscesses: 1/59 (1.7%) during cecal perforation, 1/59 (1.7%) during sigmoid perforation, and 1/59 (1.7%) occurring days after endoscopic removal of the PP in the sigmoid colon. Other severe complications included fistula formation in 7/59 (11.8%) cases: 3/59 (5.1%) coloduodenal fistula with ascending colon perforation, 2/59 (3.4%) colovesical fistulas after sigmoid perforation, 1/59 (1.7%) colovaginal fistula after sigmoid perforation, and 1/42 (1.7%) colocutaneous fistula after ascending colon perforation, treated by manual removal of the PP. Additionally, double perforation occurred in 3/59 (5.1%) patients due to PP impaction in the sigmoid diverticula (2) and sigmoid (1).

Treatment: Treatment was surgical, endoscopic, combined (endoscopy+surgery), or manual in 42/59 (71.2%), 14/59 (23.7%), 2/59 (3.4%), and 1/59 (1.7%) cases, respectively. For the combined treatment, in 1/59 (2.3%) patients, the PP was removed endoscopically, but the presence of a pelvic abscess required surgical intervention to close the orifice. Another case involved endoscopic PP removal followed by the development of a pelvic abscess days later, which required surgical treatment. Among the 14 endoscopic treatments, in 4/16 (25%) cases, the endoscopic removal of the PP was followed by the placement of metallic clips to close the perforated diverticular orifice (Table 2).

**Table 2:** Clinical features of colon perforation by migrated biliary PP. *After the endoscopic removal, the patient developed a diverticular perforation and required an emergency laparotomy with sigmoid resection. **The patient died after surgery. PP, plastic prostheses

N	Author, publication year	Migration time (days)	Perforation (site)	Treatment
1	Ruffolo TA et al., 1992 [17]	365	Sigmoid diverticulum	Endoscopy removal
2	Ruffolo TA et al., 1992 [17]	60	Sigmoid diverticulum	Endoscopy removal
3	D’Costa H et al., 1994 [20]	90	Ascending colon	Surgery
4	Schaafsma RJ et al., 1996 [21]	180	Sigmoid diverticulum	Surgery
5	Baty V et al., 1996 [22]	21	Sigmoid diverticulum	Surgery
6	Lenzo NP and Garas G, 1998 [23]	30	Sigmoid diverticulum	Surgery
7	Ang BK et al., 1998 [24]	1095	Coloduodenal fistula (ascending colon)	Surgery
8	Storkson RH et al, 2000 [25]	14	Sigmoid	Surgery
9	Figueras RG et al., 2001 [26]	14	Colocutaneous fistula (ascending colon)	Manual removal
10	Klein U et al., 2001 [27]	1095	Sigmoid diverticulum	Surgery
11	Wilhelm A et al., 2003 [28]	730	Colovesical fistula (sigmoid)	Surgery
12	Elliott M et al., 2003 [29]	120	Sigmoid	Surgery
13	Diller R et al., 2003 [30]	38	Pelvic abscess (sigmoid diverticulum)	Endoscopy removal + surgery*
14	Blake AM et al., 2004 [31]	90	Colovaginal fistula (sigmoid diverticulum)	Surgery
15	Soto-Avila JJ et al., 2006 [32]	540	Colovesical fistula (sigmoid diverticulum)	Surgery
16	Anderson EM et al., 2007 [33]	60	Pelvic abscess (sigmoid)	Endoscopy removal
17	Namdar T et al., 2007 [34]	540	Rectum	Surgery
18	Belyaev O et al., 2008 [35]	21	Sigmoid diverticulum (double perforation)	Surgery
19	Aryal KR et al., 2008 [36]	90	Sigmoid diverticulum	Surgery
20	Hunter K et al., 2010 [12]	2190	Sigmoid diverticulum	Surgery
21	Bagul A et al., 2010 [37]	30	Sigmoid diverticulum	Endoscopy removal
22	Wagemakers S et al., 2011 [38]	30	Sigmoid	Surgery
23	Peter A et al., 2011 [39]	150	Sigmoid diverticulum	Surgery
24	Jafferbhoy SF et al., 2011 [40]	90	Sigmoid diverticulum	Endoscopy removal
25	Lankisch TO et al., 2011 [41]	60	Sigmoid	Surgery
26	Malgras B et al, 2011 [42]	15	Sigmoid diverticulum (double perforation)	Surgery
27	Alcaide N et al., 2012 [43]	15	Sigmoid diverticulum	Endoscopy removal
28	Kittappa K et al., 2013 [44]	540	Sigmoid diverticulum	Surgery
29	Jones M et al., 2013 [45]	540	Pelvic abscess (caecum)	Endoscopy removal + surgery
30	de Andres AB et al., 2013 [13]	60	Sigmoid diverticulum	Surgery
31	Warnock JM et al., 2013 [14]	60	Sigmoid diverticulum	Surgery **
32	Barut I and Tarhan OR, 2014 [46]	330	Caecum	Surgery
33	Konstantinidis C et al., 2014 [47]	60	Sigmoid (double perforation)	Surgery
34	Virgilio E et al., 2015 [18]	N/A	Sigmoid	Surgery
35	Virgilio E et al., 2015 [18]	N/A	Sigmoid	Endoscopy removal
36	Mady RF et al., 2015 [48]	30	Sigmoid	Surgery
37	Chittleborough TJ et al., 2016 [49]	30	Sigmoid	Surgery
38	Chou ND et al., 2017 [50]	N/A	Sigmoid	Endoscopy removal + clip closure
39	Siaperas P et al., 2017 [51]	180	Sigmoid	Surgery
40	Hogendorf P et al., 2018 [15]	180	Sigmoid diverticulum	Surgery
41	Hogendorf et al., 2018 [15]	30	Rectum	Surgery
42	Riccardi M et al., 2019 [52]	28	Sigmoid	Surgery
43	Hnaris K and Bechara R, 2019 [53]	N/A	Ascending colon	Endoscopy removal + clip closure
44	Marcos P et al., 2020 [54]	30	Sigmoid	Surgery
45	Tao Y and Long J, 2021 [55]	90	Sigmoid	Surgery
46	Pengemä P et al., 2021 [56]	4	Appendix	Surgery
47	Park TY et al., 2021 [57]	30	Ascending colon diverticulum	Surgery
48	Ong XZ and Leow Y, 2021 [16]	2920	Caecum	Surgery
49	Yamaguchi D et al., 2022 [58]	30	Sigmoid diverticulum	Endoscopy removal + clip closure
50	Kodia K et al., 2022 [59]	60	Ascending colon	Surgery
51	Kwong E et al., 2023 [60]	N/A	Sigmoid	Endoscopy removal
52	Rybinski F et al., 2023 [61]	30	Ascending colon	Endoscopy removal + clip closure
53	Mohammadi Tofigh A et al., 2023 [62]	1095	Caecum	Surgery
54	Berdugo Hurtado F et al., 2023 [63]	120	Coloduodenal fistula (ascending colon)	Endoscopy removal
55	He Y et al., 2024 [64]	60	Coloduodenal fistula (ascending colon)	Surgery
56	Vergara-Fernándes O et al., 2024 [65]	43	Ascending colon	Surgery
57	Swied MY et al., 2024 [66]	N/A	Appendix	Surgery
58	Beloy JB et al., 2024 [67]	11	Sigmoid	Surgery
59	Ardengh AO et al., 2025	180	Sigmoid diverticulum	Endoscopy removal

## Discussion

The insertion of a PP in the CBD is a well-established therapeutic strategy for the treatment of obstructive jaundice caused by benign and malignant diseases [1,2]. Complications are rare and may occur either during the insertion of the PP or subsequently [3]. Early complications include AP, infection, and bleeding. Inadvertent manipulation of the CBD and/or the main pancreatic duct during catheterization, sphincterotomy, or the use of contrast agents activates pancreatic coenzymes, triggering an inflammatory process that may lead from minimal to severe systemic complications [4]. Late complications related to the use of PP include dysfunction, obstruction, migration, AC, bleeding, intestinal obstruction, ulceration, and perforation of the small intestine or colon, as well as other organs, resulting in abscesses and/or fistulas [3-5], as observed in the patient of the current case report.

The PP migration occurs in approximately 6% of cases [2]. Some studies report incidence rates of proximal and distal migration of biliary and pancreatic PP ranging from 4.9% to 7.5% [2,3]. Distal migration of PP is more frequent in benign strictures compared to those caused by malignant diseases, similar to what occurred with our patient [2,3].

PP dysfunction is frequently delayed in diagnosis and is significant. The migration of the PP can occur proximally or distally. Obstruction of the PP is caused by "dirty" or thick bile, biliary sludge, bacterial biofilm, or tumor growth through or around the PP [2-4]. This complication is much more common in PP compared to SEMS, especially in smaller-diameter PP.

Some authors believe that adherence caused by tumor tissue decreases PP mobility within the CBD, thereby reducing the likelihood of migration. The insertion of PP reduces the local inflammatory process found in benign conditions, decreasing PP adherence and facilitating its displacement. Other authors have reported that malignant diseases more effectively anchor the PP, acting as a rack, causing its displacement, particularly toward the proximal portion of the biliary tract. The implantation of a single, short, and thick PP also favors displacement [3-6].

Endoscopic treatment of migrated PP is feasible in 90% of cases with low morbidity [2]. When follow-up imaging does not identify the PP in the CBD and migration toward the intestine is confirmed, PP retrieval depends on its location. Usually, the PP is excreted in feces without major issues. However, when it remains impacted for an extended period, it can lead to serious and potentially life-threatening complications. Most complications due to distal migration occur in the duodenum. In cases of partial migration involving a long PP, friction against the duodenal wall opposite to the papilla may result in ulceration, which can progress to perforation [8,18,49]. Abdominal diseases such as inflammatory bowel strictures, internal hernias, and diverticular disease of the colon are risk factors for PP impaction, increasing the risk of intestinal loop perforation, similar to the patient in the current case report [6,8]. Following distal migration, PP can be removed via colonoscopy, especially in the absence of colon perforation. However, in cases of potential perforation, a detailed assessment of the patient's clinical condition and imaging findings guides the best treatment approach, whether endoscopic or surgical. The absence of peritonitis, sepsis, hemodynamic instability, and the involvement of a trained surgical team allows for the endoscopic removal of PP without subsequent complications, as demonstrated within the case report [17,18,30,33,37,40,43,45,50,53,58,60,61,63].

A review of the literature identified 58 cases of distally migrated PP with colon perforation [12-18,20-67]. Most patients were elderly, with a mean age of 69.7 years, and a higher prevalence in women (40/59 (68%)), as observed in our patient. Most cases involved benign diseases (50 out of 59 (85%)) affecting the biliary tract, while malignant diseases were present in nine out of 59 (15%) of cases. Choledocholithiasis (36/59 (61%)) was the most prevalent finding, similar to our case, followed by bile duct strictures (14/59 (23.7%)), biliary fistulas post-laparoscopic cholecystectomy (3/59 (6%)), and giant juxtapapillary diverticulum with CBD dilation (1/59 (2%)). PC, primary pancreatic lymphoma, and gallbladder carcinoma were identified in 7/59 (12%), 1/59 (2%), and 1/59 (2%) of cases, respectively.

Difficulty clearing the CBD due to calculi, leading to the option of a PP implant, was frequently reported in 23 patients found in the literature [22], similar to our case. As in our patient, the 10F, 10 cm PP was the most frequently implanted (18.6%) among all patients, indicating that large diameter and length do not prevent distal PP migration. Notably, the time for PP migration ranged from four days to eight years, with an average of 274.4 days (4-2920). Our patient presented symptoms 180 days after PP insertion via ERCP for the treatment of large CBD stones.

In most cases, the exact time of displacement, impaction, and intestinal perforation could not be estimated due to a lack of clinical follow-up. The preferred site for PP impaction was the sigmoid colon in 41/59 (69.4%), followed by the ascending colon in 10/59 (17%). Diverticular disease of the colon was the most significant risk factor, present in 23/59 (39%) and 1/59 (1.7%) of the sigmoid and ascending colon cases, respectively. These risk factors were also present in our patient.

Major complications included pelvic abscesses in 3/59 (5%) and fistulas in 7/59 (11.8%), such as colovesical in 2/59 (4.6%), coloduodenal in 3/59 (5%), colovaginal in 1/59 (1.7%), and colocutaneous in 1/59 (1.7%) fistulas. Most patients presented with abdominal pain; two exhibited urinary symptoms, including recurrent infections, pneumaturia, and dysuria [22,25]. Among these, one had fecal leakage into the vagina [23], one had cutaneous drainage of secretions [8], and another presented with osteoarticular symptoms due to a pelvic abscess, similar to what occurred in this case report. All were managed surgically.

Treatment included surgical, endoscopic, combined (endoscopy + surgery), and manual PP removal approaches in 42/59 (71.2%), 14/59 (23.7%), 2/59 (3.4%), and 1/59 (1.7%) cases, respectively. In four endoscopic cases (25%), metallic clips were placed to close the perforated diverticular orifice after PP removal. In our case, this approach was unnecessary as computed tomography already showed the area around the PP to be blocked. In addition, the mortality rate resulting from postoperative complications in this study was 1/59 (1.7%) [14]. This rate is below that described in the systematic review by Wilson et al, which was 17.4% [9].

## Conclusions

This study describes a rare and serious complication with high morbidity following distal migration of a PP used in the treatment of giant choledocholithiasis. Careful positioning of the PP during ERCP at the time of insertion and periodic follow-up after its implantation did not prevent this complication. Although distal migration of the PP can lead to perforation, it can be treated endoscopically. However, preoperative assessment of the patient's clinical condition and precise localization of the perforation are essential for the successful removal of the PP via endoscopy.
